# Using anodic aluminum oxide templates and electrochemical method to deposit BiSbTe-based thermoelectric nanowires

**DOI:** 10.1186/1556-276X-9-63

**Published:** 2014-02-07

**Authors:** Hsin-Hui Kuo, Chin-Guo Kuo, Chia-Ying Yen, Cheng-Fu Yang

**Affiliations:** 1 Department of Electrical Engineering, National University of Kaohsiung, Kaohsiung 811, Taiwan; 2 Department of Industrial Education, National Taiwan Normal University, Taipei 106, Taiwan; 3 Department of Chemical and Materials Engineering, National University of Kaohsiung, Kaohsiung 811, Taiwan

**Keywords:** Thermoelectric, Cyclic voltammetry, Electrolyte formula, Nanowires

## Abstract

In this study, the cyclic voltammetry method was first used to find the reduced voltages and anodic peaks of Bi^3+^, Sb^3+^, and Te^4+^ ions as the judgments for the growth of the (Bi,Sb)_2 - *x*_ Te_3 + *x*_-based materials. Ethylene glycol (C_2_H_6_O_2_) was used as a solvent, and 0.3 M potassium iodide (KI) was used to improve the conductivity of the solution. Two different electrolyte formulas were first used: (a) 0.01 M Bi(NO_3_)_3_-5H_2_O, 0.01 M SbCl_3_, and 0.01 M TeCl_4_ and (b) 0.015 M Bi(NO_3_)_3_-5H_2_O, 0.005 M SbCl_3_, and 0.0075 M TeCl_4_. The potentiostatic deposition process was first used to find the effect of reduced voltage on the variation of compositions of the (Bi,Sb)_2 - *x*_Te_3 + *x*_-based materials. After finding the better reduced voltage, 0.01 M Bi(NO_3_)_3_-5H_2_O, 0.01 M SbCl_3_, and 0.01 M TeCl_4_ were used as the electrolyte formula. The pulse deposition process was successfully used to control the composition of the (Bi,Sb)_2 - *x*_Te_3 + *x*_-based materials and grow the nanowires in anodic aluminum oxide (AAO) templates.

## Background

Thermoelectric energy conversion has attracted much interest as a possible application for environmentally friendly electric-power generators and highly reliable, accurate temperature-controllable refrigerators used as electronic devices because it is one of the simplest technologies applicable to energy conversion [1-4]. The efficiency of thermoelectricity is governed by a basic property of thermoelectrical material, and the figure of merit of a thermoelectric material is defined by

(1) $$\mathrm{ZT}=S^{2}T\;\delta /\left(\kappa_{e}+\kappa_{l}\right),$$

where *T* is the absolute temperature. As Equation 1 shows, optimally thermoelectric materials will have high electrical conductivity (*δ*), low thermal conductivity (the electron thermal conductivity *κ*_e_ and the lattice thermal conductivity *κ*_l_), and a high thermoelectric power (*S*, Seebeck coefficient). For a material to be a good thermoelectric cooler, it must have a high thermoelectric figure of merit ZT. Much of the recent work on thermoelectric materials has focused on the ability of heterostructures and quantum confinement to increase efficiency over bulk materials [5-7].

So far, the thermoelectrical materials used in applications have all been in bulk (3D) and thin film (2D) forms. However, Hicks et al. had pointed out that low-dimensional materials (for example 1D for nanowires) have better efficiency than bulk and thin film forms due to low-dimensional effects on both charge carriers and lattice waves [8]. However, since the 1960s, only slow progress has been made in enhancing ZT [9], either in BiSbTe-based alloys or in other thermoelectric material. The validity of attaining higher ZT value in low dimension systems has been experimentally demonstrated on Bi_2_Te_3_/Sb_2_Te_3_ superlattices [10] and on PbTe/PbSeTe quantum dots [2] with ZT of approximately 2.4 and 1.6, respectively, at 300 K. Therefore, nanowires are potentially good thermoelectrical systems for application. In the past, electrochemical deposition was a useful method to deposit the materials in different morphologies, including thin films and nanowires [11].

The successfully practical applications of the nanostructured thermoelectric devices must investigate a cost-effective and high-throughput fabrication process. In the past, many various techniques, including chemical vapor deposition [10], molecular beam epitaxy [12], vapor-liquid-solid growth process [13], and hydrothermal process [14], had been applied to synthesize nanowire-, nanotube-, or thin film-structured thermoelectric materials. Compared to those methods, electrodeposition is one the most cost-effective techniques to fabricate the nanostructured materials [15]. In this study, commercial honeycomb structure anodic aluminum oxide (AAO) nanotube arrays were used as the templates, and the cyclic voltammetry process was used as the method to deposit the (Bi,Sb)_2 - *x*_Te_3 + *x*_-based thermoelectric nanowires. At first, potentiostatic deposition process and two different electrolyte formulas were used to find the effects of ionic concentrations on the composition fluctuation of the deposited (Bi,Sb)_2 - *x*_Te_3 + *x*_ materials. After finding the better deposition parameters, AAO thin films with a nanotube structure were used a template to fabricate the (Bi,Sb)_2 - *x*_Te_3 + *x*_ nanowires by means of the pulse deposition process. We would show that the (Bi,Sb)_2 - *x*_Te_3 + *x*_ nanowires with (Bi + Sb)/Te atomic ratio close to 2/3 could be successfully grown.

## Methods

**Figure 1:**
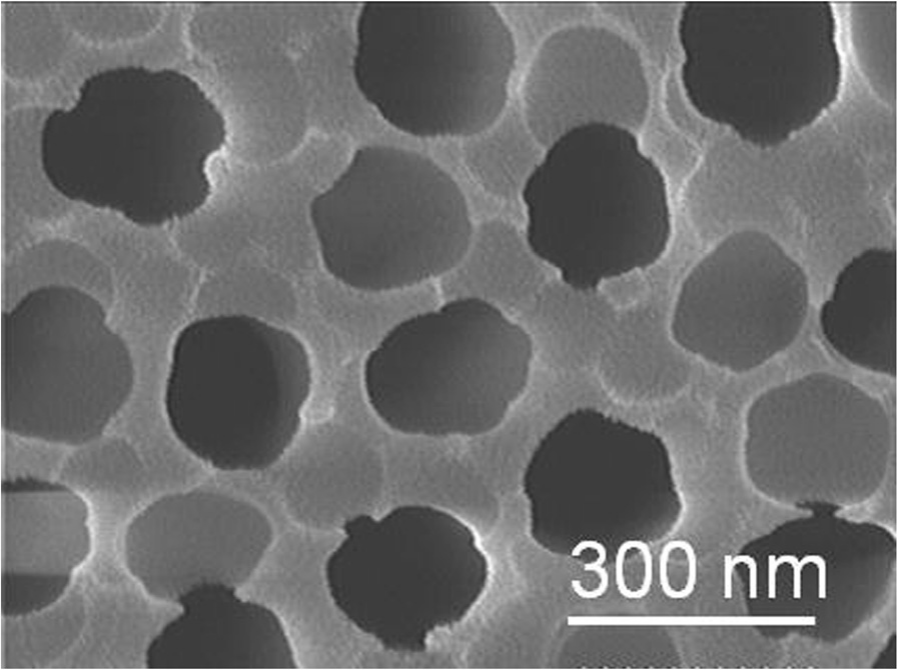
SEM morphology of the AAO templates.

For the AAO templates, an annealed high-purity (99.99%) aluminum foil was electropolished in a mixture of HClO_4_ (25% in volume ratio) and C_2_H_5_OH (75%) until the root mean square surface roughness of a typical 10 μm × 10 μm area was 1 nm. In this study, a two-step electrochemical anodization was used to fabricate AAO template. For the first anodization process, the foil was anodized in 10% sulfuric acid (H_2_SO_4_) and 3% oxalic acid (H_2_C_2_O_4_) at 25°C at a constant voltage of 40 V for 60 min, using to obtain AAO substrates with nanotube arrays of self-organized honeycomb structure [16]. Then a semi-finished AAO was produced, and subsequently the thick oxide was stripped away by immersing the Al sample in a mixture of 2 wt.% chromic acid and 6 wt.% phosphoric acid at 60°C. The second anodization process, which was similar to the first stage, was carried out until the remaining Al sample was completely anodized, and a finished AAO template was thus fabricated [17]. Nevertheless, we further widened the pores of nanotubes by using a 5 wt.% phosphoric acid solution at 25°C for 30 min. The resulting thickness of the AAO templates was about 70 μm. The cylindrical nanotubes penetrated the entire thickness of the AAO templates. As Figure 1 shows, the hole diameter of each tube was approximately 250 nm and the hole wall of each tube was around 60 to 100 nm.

Two different concentrations of electrolyte formula, (a) 0.01 M Bi(NO_3_)_3_-5H_2_O, 0.01 M SbCl_3_, and 0.01 M TeCl_4_ and (b) 0.015 M Bi(NO_3_)_3_-5H_2_O, 0.005 M SbCl_3_, and 0.0075 M TeCl_4_, were first used to find the effects of ionic concentrations on the composition fluctuation of the reduced (Bi,Sb)_2 - *x*_Te_3 + *x*_ materials by using the potentiostatic deposition process. After finding the better deposition parameters, AAO thin films had a nanotube structure and could be used as a template to fabricate the nanowire materials. In order to proceed the (Bi,Sb)_2 - *x*_Te_3 + *x*_ materials, ethylene glycol (C_2_H_6_O_2_) was used as an solvent and 0.3 M potassium iodide (KI) was used to improve the conductivity of the solution. Deposition of (Bi,Sb)_2 - *x*_Te_3 + *x*_ nanowires in AAO templates was investigated by means of the pulse deposition process by using the C_2_H_6_O_2_ solvent containing 0.3 M KI, 0.015 M Bi(NO_3_)_3_-5H_2_O, 0.005 M SbCl_3_, and 0.0075 M TeCl_4_. The morphologies of the deposited (Bi,Sb)_2 - *x*_Te_3 + *x*_ compositions were observed using field-emission scanning electron microscope (FESEM), and energy dispersive spectroscopy (EDS) was used to analyze the deposited (Bi,Sb)_2 - *x*_Te_3 + *x*_ compositions.

## Results and discussion

At the first, we use the cyclic voltammetry experiment that the working electrode potential is linearly ramped versus time like linear sweep voltammetry, and the experiment's scan rate is 10 mV/s and the scan range is 0.4 to -0.7 V. When only the pure C_2_H_6_O_2_ solvent was used as solution, the current peak for the reduced and oxidized reaction was not observed (not shown here). This result proves that the C_2_H_6_O can be used as the solvent, and it will not influence the results of the cyclic voltammetry deposition. When only the 0.3 M KI was used as electrolyte formula, the current peak for the reduced and oxidized reactions were not observed (not shown here) in the range of 0.20 to -0.80 V. As the voltage was in the range of 0.20 to 0.40 V, the oxidized current increased. This oxidized reaction is believed to be caused by I^-^ oxidized into I_2_, as the following (Equation 2):

(2) $$2I^{–}\to I_{2}+2e^{–}$$

**Figure 2:**
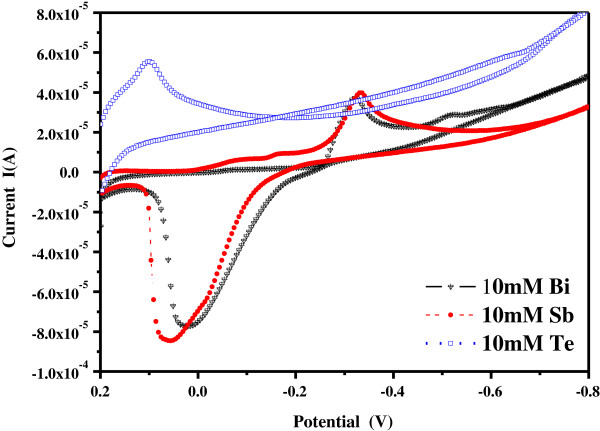
**Cyclic voltammetry curves of the Bi**^**3+**^**, Sb**^**3+**^**, and Te**^**4+**^**in ethylene glycol.**

Figure 2 shows the cyclic voltammetry curves of the Bi^3+^, Sb^3+^, or Te^4+^ ions, only the 0.01 M Bi(NO_3_)_3_-5H_2_O, 0.01 M SbCl_3_, and 0.01 M TeCl_4_ each alone was added into pure ethylene glycol as electrolyte formula. Figure 2 shows that the reduced reactions of Bi^3+^, Sb^3+^, and Te^4+^ ions shown in Equations 3 to 5 started at -0.23, -0.23, and 0.20 V, respectively:

(3) $${\mathrm{Bi}}^{3+}+3e^{–}\to \mathrm{Bi}$$

(4) $${\mathrm{Sb}}^{3+}+3e^{–}\to \mathrm{Sb}$$

(5) $${\mathrm{Te}}^{4+}+4e^{–}\to \mathrm{Te}$$

The cyclic voltammetry curves suggest that Te is the first metal that will be reduced. Bi^3+^ and Sb^3+^ have the same reduced voltage range and the reduced voltage peaks for Bi^3+^ and Sb^3+^ ions are -0.325 and -0.334 V, respectively. Because the voltage in the range of 0.20 to -0.80 V is used, the voltage will not reduce 2I^–^ ions into I_2_. The EDS analysis also shows that the iodine is not detected in the reduced (Bi,Sb)_2 - *x*_Te_3 + *x*_-based materials (will be proven in analyzed results of Tables 1 and 2). Those results prove that the addition of 0.3 M KI will not influence the reduced results of the Bi^3+^, Sb^3+^, and Te^4+^ ions.

**Table 1:** **Effects of deposition voltage of the potentiostatic deposition process on the compositions of the (Bi,Sb)**_**2 -**__***x***_**Te**_**3**__**+**__***x***_**materials**

**Potential (V)**	**Electrolyte formula (a)**			**Electrolyte formula (b)**		
	**Atomic ratio (%)**			**Atomic ratio (%)**		
	**Sb**	**Te**	**Bi**	**Sb**	**Te**	**Bi**
0.00	0.00	94.50	5.50	1.48	92.16	6.36
-0.20	5.32	89.22	5.54	6.88	68.86	24.26
-0.30	37.35	44.05	18.61	7.42	35.14	57.43
-0.40	36.23	44.01	19.78	9.97	30.19	59.83
-0.50	41.42	33.72	24.86	10.57	27.46	61.97
-0.60	45.15	44.75	10.11	11.83	29.48	58.69

Effects of deposition voltage of the potentiostatic deposition process on the compositions of the (Bi,Sb)_2 - *x*_Te_3 + *x*_ materials, and deposition time was 60 min. Electrolyte formula was (a) 0.01 M Bi(NO_3_)_3_-5H_2_O, 0.01 M SbCl_3_, and 0.01 M TeCl_4_ and (b) 0.015 M Bi(NO_3_)_3_-5H_2_O, 0.005 M SbCl_3_, and 0.0075 M TeCl_4_, respectively.

**Table 2:** **Effects of** ***t***_**off**_**in pulse deposition process on the compositions of (Bi,Sb)**_**2 -**__***x***_**Te**_**3 +**__***x***_**materials**

	**Sb**	**Te**	**Bi**
Potentiostatic deposition process	9.97	30.19	59.83
*t*_off_ = 0.1 s	7.09	31.29	61.63
*t*_off_ = 0.4 s	7.71	51.25	41.05
*t*_off_ = 1 s	12.02	69.43	18.54
*t*_off_ = 1.6 s	7.22	79.62	13.16
*t*_off_ = 2 s	5.77	84.06	10.17
*t*_off_ = 4 s	6.24	86.30	7.46

The electrolyte formula was 0.015 M Bi(NO_3_)_3_-5H_2_O, 0.005 M SbCl_3_, and 0.0075 M TeCl_4_; the bias voltage was set at -0.4 V; *t*_on_ was set at 0.2 s; and *t*_off_ was changed from 0.1 to 4 s.

**Figure 3:**
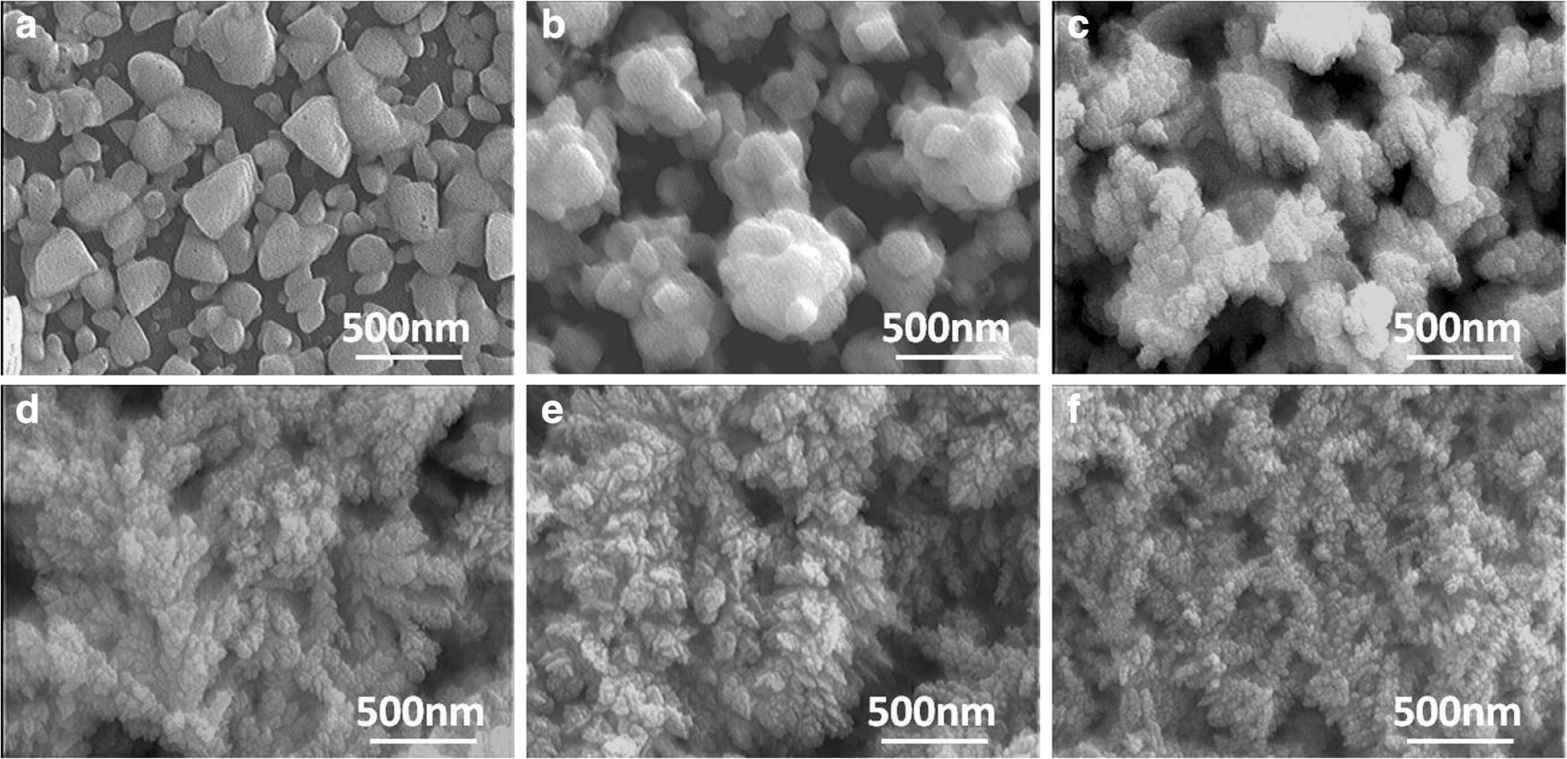
**SEM micrographs of formula 0.01 M Bi(NO**_**3**_**)**_**3**_**-5H**_**2**_**O, 0.01 M SbCl**_**3**_**, and 0.01 M TeCl**_**4**_**.** SEM micrographs of the electrolyte formula 0.01 M Bi(NO_3_)_3_-5H_2_O, 0.01 M SbCl_3_, and 0.01 M TeCl_4_, as a function of reduced voltage **(a)** 0 V, **(b)** -0.2 V, **(c)** -0.3 V, **(d)** -0.4 V, **(e)** -0.5 V, and **(f)** -0.6 V.

In order to discuss the effects of electrolyte concentrations on the fluctuation of the reduced (Bi,Sb)_2 - *x*_Te_3 + *x*_ compositions, two different electrolyte formulas were first used: (a) 0.01 M Bi(NO_3_)_3_-5H_2_O, 0.01 M SbCl_3_, and 0.01 M TeCl_4_ and (b) 0.015 M Bi(NO_3_)_3_-5H_2_O, 0.005 M SbCl_3_, and 0.0075 M TeCl_4_. The potentiostatic deposition process was first used to deposit the (Bi,Sb)_2 - *x*_Te_3 + *x*_ materials. Figure 3 shows the scanning electron microscopy (SEM) images of the electrolyte formula 0.01 M Bi(NO_3_)_3_-5H_2_O, 0.01 M SbCl_3_, and 0.01 M TeCl_4_, as a function of reduced voltage (0.00 V and -0.20 to -0.60 V). From the morphology of Figure 3, as the reduced voltage was changed from 0.00 to -0.20 V, the deposited materials changed from disk-typed particles with dispersant structure to a nanoparticle-aggregated structure, as Figure 3a,b shows. We will show in Table 1 that the main element in the disk-typed particles and nanoaggregated particles is Te. The average diameters of the particle sizes shown in Figure 3a,b were 180 and 320 μm, respectively. As the reduced voltage was shifted to more negative (-0.30 to -0.60 V), the deposited materials obtained by the cyclic voltammetry process were grown into branch-typed particles, and their particle sizes were really in the nanoscale (nanometer), as Figure 3c,d,e,f shows.

**Figure 4:**
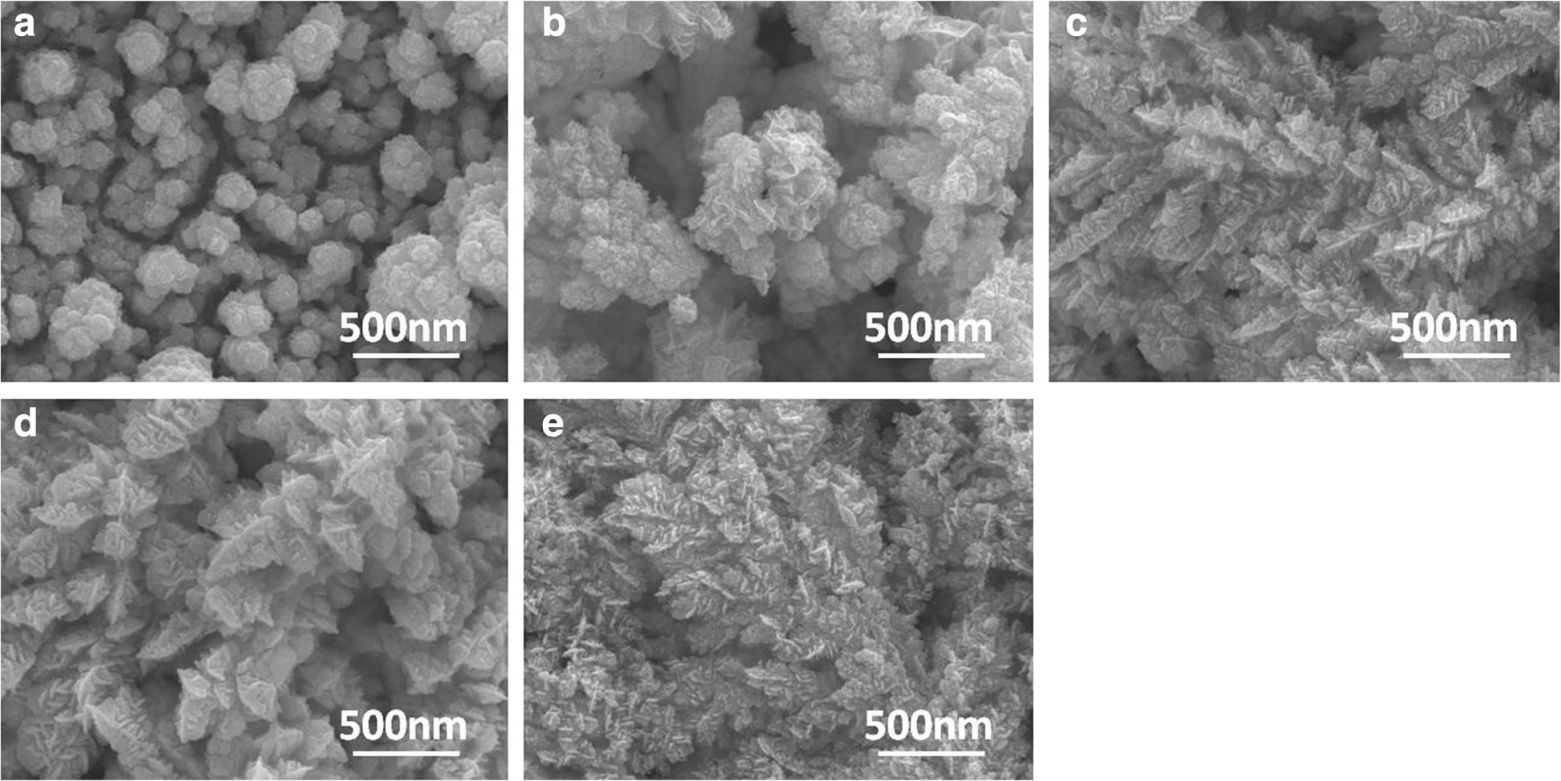
**SEM micrographs of formula 0.015 M Bi(NO**_**3**_**)**_**3**_**-5H**_**2**_**O, 0.005 M SbCl**_**3**_**, and 0.0075 M TeCl**_**4**_**.** SEM micrographs of the electrolyte formula 0.015 M Bi(NO_3_)_3_-5H_2_O, 0.005 M SbCl_3_, and 0.0075 M TeCl_4_, as a function of reduced voltage **(a**) -0.2 V, **(b)** -0.3 V, **(c)** -0.4 V, **(d)** -0.5 V, and **(e)** -0.6 V.

Figure 4 shows the SEM micrographs of the electrolyte formula 0.015 M Bi(NO_3_)_3_-5H_2_O, 0.005 M SbCl_3_, and 0.0075 M TeCl_4_, as a function of reduced voltage (-0.20 to -0.60 V). Figure 4 also shows that as the reduced voltage was changed from 0.00 V (not shown here) to -0.20 V; as Figure 4a shows, the deposited materials changed from disk-typed particles to nanoaggregated particles. The average diameters of the particle sizes shown in Figure 4a were 130 μm. As the reduced voltage was shifted to -0.30 to -0.60 V, the deposited materials obtained by the cyclic voltammetry process were really in the nanoscale (nanometer), as Figure 4b,c,d,e shows. As compared to the results in Figures 3 and 4, the reduced voltage in the range of 0.00 to -0.20 V is not suitable to deposit the nanowires, because the main composition is Te (will be proven in Table 1) and the process leads large particle aggregation.

Table 1 shows the effects of different deposition voltages on the compositions of the deposited materials, and deposition time was 60 min. The results in Table 1 show that as the voltage was in the range of 0.00 to -0.20 V, the main element is the deposited Te. The (Bi,Sb)_2 - *x*_Te_3 + *x*_ compositions were obtained as the voltage in the range of -0.20 to -0.60 V. The results in Table 1 reveal that the electrolyte formula and the deposition voltage are the two important parameters to influence the (Bi,Sb)_2 - *x*_Te_3 + *x*_ compositions. Table 1 also shows that the two different electrolyte formulas have the same variation trends as the used voltage increases. As the voltage was changed from 0.00 to -0.50 V, the ratios of Bi and Sb elements in (Bi,Sb)_2 - *x*_Te_3 + *x*_ compositions increased. Two reasons are believed to cause those results. First, the reduced reactions of Bi^3+^, Sb^3+^, and Te^4+^ ions start at -0.23, -0.23, and 0.20 V (Figure 2). For that, as 0.00 to -0.20 V is used, the main element in the deposited materials is Te. As the voltage is smaller than -0.30 V, the driving forces of reduction for Bi^3+^ and Sb^3+^ ions increase and the ratios of Bi and Sb elements in the deposited compositions increase. Second, the driving force for mass transfer is typically a difference in chemical potential, though other thermodynamic gradients may couple to the flow of mass and drive it as well. As the voltage value is more negative (means the applied voltage is larger than the needed reduction voltage), the mass transfer effect will influence the compositions of the deposited (Bi,Sb)_2 - *x*_Te_3 + *x*_ materials. A chemical species moves from areas of high chemical potential to areas of low chemical potential. Thus, the maximum theoretical extent of a given mass transfer is typically determined by the point at which the chemical potential is uniform.

For multiphase systems, chemical species will often prefer one phase over the others and reach a uniform chemical potential only when most of the chemical species has been absorbed into the preferred phase, while the actual rate of mass transfer will depend on additional factors including the flow patterns within the system and the diffusivities of the species in each phase. As shown in Table 1, because the Te^4+^ ions have lower concentration in the two electrolyte formulas, it will easily reach the mass transfer condition because of higher consumption and then Te^4+^ ions will reach a saturation value (about 44 at.% for electrolyte formula (a) and 30 at.% for electrolyte formula (b)) even larger negative voltage is used. As compared for Bi^3+^ and Sb^3+^ ions, they have the larger negative reduced voltage and lower consumption, the mass transfer effect will not happen. For that, the concentrations of Bi and Sb elements will increase with increasing bias voltage (large negative voltage).

When the potentiostatic deposition process is used, the obtained results prove that as more negative voltage is used as bias, the electrolyte concentrations (or ion diffusion effect) will influence the compositions of the deposited (Bi,Sb)_2 - *x*_Te_3 + *x*_ materials. If we control the diffusion of ions (Bi^3+^, Sb^3+^, and Te^4+^), we can regulate the compositions of the deposited (Bi,Sb)_2 - *x*_Te_3 + *x*_ materials. For that, the pulse deposition process is used to deposit the electrolyte formula of 0.015 M Bi(NO_3_)_3_-5H_2_O, 0.005 M SbCl_3_, and 0.0075 M TeCl_4_. The bias voltage was set at -0.40 V, the bias on time (*t*_on_) was set at 0.2 s, and the duration of off time (*t*_off_) was changed from 0.1 to 4 s, respectively. The EDS-analyzed results are compared in Table 2 as a function of duration of off time (*t*_off_), and the atom ratio of Te in the deposited (Bi,Sb)_2 - *x*_Te_3 + *x*_ materials increased. As the duration of *t*_off_ was 0.2 s, the (Bi + Sb)/Te atomic ratio was larger than 2/3; as the duration of *t*_off_ was in the range of 0.4 to 1 s, the (Bi + Sb)/Te atomic ratio was close to 2/3; as the duration of *t*_off_ was longer than 1 s, the Te atomic ratio was larger than 70%.

Those results can be explained by the characteristics of the potentiostatic deposition process. As the duration of *t*_off_ is 0.2 s, the diffusion layer (the variation in the concentrations of Bi^3+^, Sb^3+^, and Te^4+^ ions) is formed. Apparently, in the duration of *t*_off_, the consumed Te^4+^ ions are compensated and the effect of mass transfer will decrease in the deposition process. Also, the reduced voltage of Te^4+^ ions is 0.20 V; for that, the deposition concentration of Te increases with increasing duration of *t*_off_. The effect of mass transfer on Bi^3+^ and Sb^3+^ ions is smaller than on Te^4+^ ions; for that, the deposition concentrations of Bi and Sb will not increase with increasing duration of *t*_off_. Undoubtedly, the pulse deposition process can control the mass transfer and then can control the compositions of the deposited (Bi,Sb)_2 - *x*_Te_3 + *x*_ materials. However, the iodine cannot be detected in the reduced (Bi,Sb)_2 - *x*_Te_3 + *x*_-based materials.

**Figure 5:**
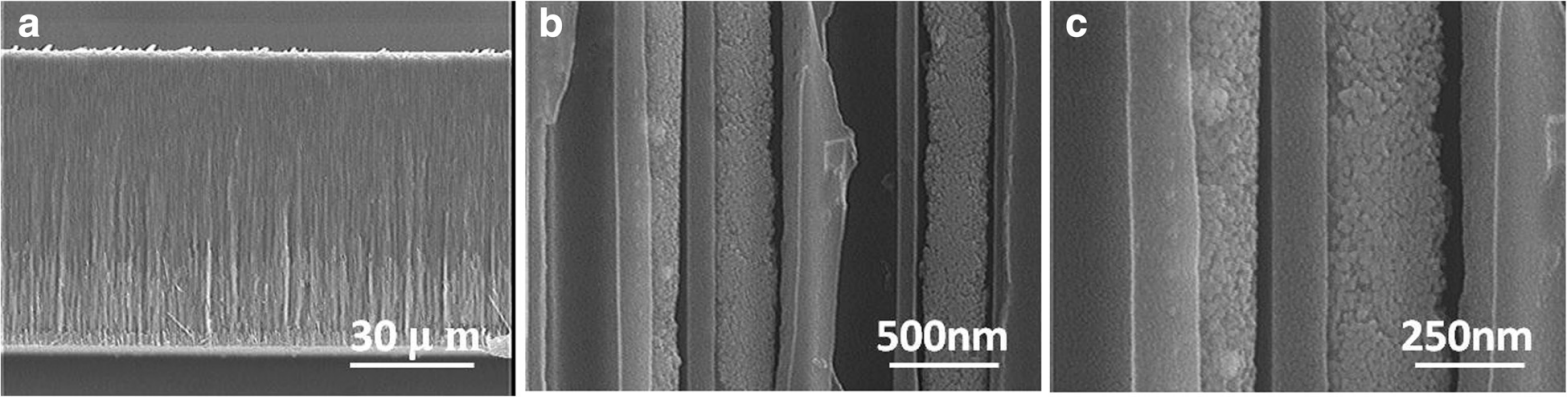
**SEM micrographs of the (Bi,Sb)**_**2 -*x***_**Te**_**3 +*x***_**-based nanowires under different magnification ratio. (a)** 1,000; **(b)** 50,000; and **(c)** 100,000. The bias voltage was set at -0.4 V, *t*_on_/*t*_off_ was 0.2/0.6 s, and the electrolyte formula was 0.015 M Bi(NO_3_)_3_-5H_2_O, 0.005 M SbCl_3_, and 0.0075 M TeCl_4_.

Finally, the electrolyte formula of 0.015 M Bi(NO_3_)_3_-5H_2_O, 0.005 M SbCl_3_, and 0.0075 M TeCl_4_ was used to fabricate the (Bi,Sb)_2 - *x*_Te_3 + *x*_-based nanowires, and the reduced voltage was -0.4 V, the *t*_on_/*t*_off_ was 0.2/0.6 s, and the cycle time was 10^5^. From the cross images shown in Figure 5, the (Bi,Sb)_2 - *x*_Te_3 + *x*_-based nanowires were successfully grown in the AAO nanotubes. As Figure 5 shows, the average length was about 28 μm, the growth rate was about 1.4 μm/h, and the diameter was about 250 nm. The atomic ratio for Bi/Sb/Te is 4.12:32.05:63.83, and the (Bi + Sb)/Te atomic ratio is more close to 2/3. When the *t*_on_/*t*_off_ was 0.2/1.0 s, the atomic ratio for Bi/Sb/Te is 3.54:22.05:74.41, and the (Bi + Sb)/Te atomic ratio is far from 2/3.

## Conclusions

In this study, the reduced reactions of Bi^3+^, Sb^3+^, and Te^4+^ started at -0.23, -0.23, and 0.20 V, and the reduced voltage peaks for Bi and Sb were -0.325 and -0.334 V, respectively. As the reduced voltage was changed from 0.00 to -0.20 V and 0.01 M Bi(NO_3_)_3_-5H_2_O, 0.01 M SbCl_3_, and 0.01 M TeCl_4_ was used as electrolyte formula, the deposited materials changed from disk-typed particles with dispersant structure to a nanoparticle-aggregated structure. As the range of 0.00 to -0.20 V was used, the main element in the deposited materials was Te. As the voltage was smaller than -0.30 V, the driving forces of reduction for Bi and Sb increased and the concentrations of Bi and Sb in the deposited compositions increased. Finally, the electrolyte formula of 0.015 M Bi(NO_3_)_3_-5H_2_O, 0.005 M SbCl_3_, and 0.0075 M TeCl_4_ in the pulse deposition process was used to deposit (Bi,Sb)_2 - *x*_Te_3 + *x*_ nanowires. As the reduced voltage was -0.4 V, the *t*_on_/*t*_off_ was 0.2/0.6 s, and the cycle time was 10^5^, the (Bi,Sb)_2 - *x*_Te_3 + *x*_-based nanowires were successfully grown in AAO templates. The nanowires had the average length of 28 μm and the diameter of about 250 nm, and the atomic ratio for Bi/Sb/Te was 4.12:32.05:63.83.

## Competing interests

The authors declare that they have no competing interests.

## Authors’ contributions

HHK and CGK proposed an idea to deposit BiSbTe-based thermoelectric nanowires and helped in the deposition of the BiSbTe-based materials. CYY participated in the experimental process and helped in the data analysis. CFY also proposed an idea to deposit BiSbTe-based thermoelectric nanowires and wrote the paper. All authors read and approved the final manuscript.
